# After-effects of repetitive transcranial magnetic stimulation with parameter dependence on long-term potentiation-like plasticity and object recognition memory in rats

**DOI:** 10.3389/fnins.2023.1144480

**Published:** 2023-09-19

**Authors:** Shanjia Chen, Xiaokuo He, XinChen Wei, Jiyi Huang, Jie Zhang

**Affiliations:** ^1^The First Affiliated Hospital of Xiamen University, Xiamen, China; ^2^Laboratory Neuropathology, Institute Medicine College, Xiamen University, Xiamen, China; ^3^Fifth Hospital of Xiamen, Xiamen, China; ^4^Xiangyang Central Hospital, Affiliated Hospital of Hubei University of Arts and Science, Xiangyang, Hubei, China; ^5^The Graduate School of Fujian Medical University, Fuzhou, Fujian, China

**Keywords:** repetitive transcranial magnetic stimulation, intensity dependence, long-term potentiation, rat hippocampus, learning and memory, novel object recognition

## Abstract

**Objective:**

To investigate the after-effects of 25-Hz repetitive transcranial magnetic stimulation (rTMS) at 60, 100, and 120% resting motor threshold (rMT) on long-term potentiation (LTP) in the rat hippocampus, to clarify the intensity dependence of rTMS, and to determine whether it simultaneously affects learning and memory ability.

**Methods:**

Five rats were randomly selected from 70 male Wistar rats, and evoked rMT potentials were recorded in response to magnetic stimulation. The remaining 65 rats were randomly assigned to five groups (*n* = 13), including sham rTMS, 1 Hz 100% rMT, and 25 Hz rTMS groups with 3 subgroups of 60% rMT, 100% rMT, and 120% rMT. Five rats in each group were anesthetized and induced by a priming TMS-test design for population spike (PS) response of the perforant path-dentate gyrus in the hippocampus; the remaining eight rats in each group were evaluated for object recognition memory in the novel object recognition (NOR) task after the different rTMS protocols.

**Results:**

Forty-five percent (approximately 1.03 T) of the magnetic stimulator output was confirmed as rMT in the biceps femoris muscle. The PS ratio was ranked as follows: 25 Hz 100% rMT (267.78 ± 25.71%) > sham rTMS (182 ± 9.4%) >1 Hz 100% rMT (102.69 ± 6.64%) > 25 Hz 120% rMT (98 ± 11.3%) > 25 Hz 60% rMT (36 ± 8.5%). Significant differences were observed between the groups, except for the difference between the 25 Hz 120% rMT and the 1 Hz 100% rMT groups (*p* = 0.446). LTP was successfully induced over the 60-min recording period only in the sham rTMS and 25 Hz 100% rMT groups. Moreover, these two groups spent more time exploring a novel object than a familiar object during the NOR task (*p* < 0.001), suggesting long-term recognition memory retention. In the between-group analysis of the discrimination index, the following ranking was observed: 25 Hz 100% rMT (0.812 ± 0.158) > sham rTMS (0.653 ± 0.111) > 25 Hz 120% rMT (0.583 ± 0.216) >1 Hz 100% rMT (0.581 ± 0.145) > 25 Hz 60% rMT (0.532 ± 0.220).

**Conclusion:**

The after-effect of 25-Hz rTMS was dependent on stimulus intensity and provided an inverted (V-shaped) bidirectional modulation on hippocampal plasticity that involved two forms of metaplasticity. Furthermore, the effects on the recognition memory ability were positively correlated with those on LTP induction in the hippocampus *in vivo*.

## 1. Introduction

Transcranial magnetic stimulation (TMS), a non-invasive transcranial brain stimulation technique, has emerged as a promising treatment for affective disorders in humans, such as depression and hypomnesia (Muellbacher et al., 2000; Wang et al., 2018). High frequencies (>5 Hz) that facilitate cortical excitability and low frequencies (≤1 Hz) that inhibit it have been applied in clinical treatment (Maeda et al., 2000; Muellbacher et al., 2000). TMS applied at 5 Hz modulated hippocampal excitation with greater encoding-retrieval similarity effects on memory representations compared with 1 Hz TMS (Wang et al., 2018). A meta-analysis demonstrated that repetitive TMS (rTMS), especially at high frequency and stimulation intensity between 80 and 120% of resting motor threshold (rMT), can improve memory function by varying degrees in patients with mild cognitive impairment when it is applied over the dorsolateral prefrontal cortex, which has strong neural connections with the hippocampus (Zhang et al., 2021). However, another meta-analysis indicated the overall effect of rTMS was negligible and statistically nonsignificant on cognitive improvement including attention and working memory (He et al., 2022). It has been reported that the effect of rTMS varies considerably in terms of stimulation intensity. Previous studies that applied rTMS consisting of up to 20 stimuli at 5, 10, or 20 Hz found frequency-dependent inhibition of motor cortical excitability at an intensity equal to the rMT, whereas cortical inhibition gradually changed to excitation at the rMT superthreshold (Modugno et al., 2001), indicating that intensity and frequency parameters were crucial to determining the after-effect of the stimulus on synaptic efficacy. Moreover, the stimulus intensity in short trains reportedly had a greater after-effect on cortical excitability than the frequency (Gilio et al., 2007). However, few studies have examined how the after-effects of rTMS on the induction of neuronal plasticity and memory are influenced by the intensity of stimulation. Understanding the mechanism that underlies this effect is crucial for clinical efficacy, and many of the details are still unknown.

Long-term potentiation (LTP) is the long-lasting increase in synaptic efficacy resulting from high-frequency stimulation of afferent fibers (Nugent et al., 2008). LTP in the hippocampus is considered a reliable model of synaptic plasticity related to the learning and memory (Cohen et al., 1999; Cooke and Bliss, 2006). The rTMS protocol was adopted to assist in rehabilitation training for the cognitive improvement (Chou et al., 2020). Neurons stabilize their synaptic transmission and adapt their intrinsic excitability in response to their prior history of synaptic or cellular activity; this process is known as the homeostatic plasticity (Delvendahl and Muller, 2019). High frequency rTMS intervention has been proved to have advantages of some efficacy and high safety for the treatment of psychobehavioral abnormalities and cognitive decline in Alzheimer’s Disease patients (Koch et al., 2018; Jiang et al., 2022). How the after-effects of the rTMS protocol for inducing hippocampal excitability are influenced by the intensity of the stimulation is not fully understood.

To clarify this issue, the present study employed a priming-test design (Karabanov et al., 2015; Muller-Dahlhaus and Ziemann, 2015), which involved a “priming” rTMS protocol that triggers a homeostatic response, followed by a “test” intermittent theta-burst stimulation (iTBS) protocol that captures the homeostatic response. iTBS is particularly effective for promoting hippocampal LTP function because this stimulation rhythm should resonate with the endogenous theta-nested-gamma activity prominent in the hippocampus (Hermiller et al., 2020). Moreover, to verify whether the hippocampal LTP induced by rTMS at different intensities (60, 100, 120% rMT) simultaneously influenced learning and memory ability, a novel object recognition (NOR) test was performed (Kang et al., 2021). We hypothesized that a priming rTMS below the rMT threshold may inhibit the response to subsequent LTP-inducing iTBS, whereas a priming rTMS above the rMT threshold may increase this response instead. In addition, we tested the hypothesis that hippocampal LTP induction was associated with an effect on learning and memory ability. This study was conducted to provide some preliminary guidance for the selection of appropriate rTMS intensity for the enhancement of hippocampal LTP and to contribute some evidence supporting the use of rTMS as a treatment of memory loss in a clinical setting.

## 2. Materials and methods

### 2.1. Ethical approval

This study was approved by the Institutional Animal Care and Use Committee of the Faculty of Medicine, Xiamen University, China (approval no. SYXK(min)-2018–0009).

### 2.2. Animals and experimental protocols

Seventy male Wistar rats weighing approximately 230 ± 10 g (approximately 6 weeks old) were obtained from the National Animal Center, Guangzhou, ZhongShan University, China. The quality certificate number for the experimental animals was 44,008,500,008,720. All the animals were fed with suitable food provided by the animal feeding center, housed at a density of four individuals per cage in a temperature-controlled room (constant 23°C ± 1°C), and maintained at a light–dark cycle of 12:12 h (lights on at 6:00 AM).

One week after arrival, five rats were selected using the random number table method, and the evoked rMT potentials in the biceps femoris muscle were recorded by magnetic stimulation. The remaining 65 rats were randomly assigned to control (sham rTMS, *n* = 13), low-frequency rTMS stimulation (1 Hz 100% rMT, *n* = 13), and high-frequency rTMS stimulation (*n* = 39) groups; the high-frequency group was divided into three subgroups: low intensity (25 Hz 60% rMT, *n* = 13), medium intensity (25 Hz 100% rMT, *n* = 13), and high intensity (25 Hz 120% rMT, *n* = 13) groups. Five rats were anesthetized in each of the five groups, and TMS was used to measure the population spike (PS) response of the perforant path-dentate gyrus (PP-DG) in the hippocampus. The remaining eight rats in each group were subjected to the NOR task to evaluate memory performance. The tests and rTMS treatment was performed in the animal experimental center from 14:00 to 21:00. If one of the subjects died or failed to complete the experimental protocol, additional animals were incorporated into the corresponding experimental group.

### 2.3. Testing apparatus

The transcranial magnetic stimulator used in the experiment (Magstim, Rapid2, UK) is a conventional, air-cooled device, with an eight-shaped coil (inner diameter 40 mm, outer diameter 90 mm) and a maximum output strength of 2.3 T.

#### 2.3.1. Electrophysiological testing equipment

Motor evoked potential (MEP) and PS were recorded using an electromyography machine (MedelecSynergy, Oxford Instruments, UK). A double-arm stereoscopic brain locator and flexible spindle craniotomy drill (Stoelting 51603, USA) were employed. Concentric bipolar electrodes were manufactured by A-M Systems (Carlsborg, WA, USA). An extracellular amplifier was applied to a single-channel recording with a filtering range of 0.1–10,000 Hz and a gain of 10 K (A-M Systems 1700, Sequim, WA, USA). The equipment comprised an analog-to-digital converter (Axon Digidata 1,440, Molecular Devices, USA), stimulation isolator (ISO-Flex, Israel), data logging and analysis software (Axon pClamp 10, Molecular Devices, USA), and urethane (ethyl carbamate, lot No. E21262, Jinyu Chemical Co., LTD., China).

#### 2.3.2. Behavioral testing equipment

The NOR task was carried out in an open box (80 cm × 80 cm × 80 cm), which was composed of black non-reflective plastic plates with an overhead camera (SONY, HDR-CX405). Soda cans with similar shapes and different colors were used as identifiers A or B.

### 2.4. A priming-test design protocol

#### 2.4.1. rMT measurement

Since determining the motor threshold in each individual rat is a stressful and invasive procedure, the five rats with the same age (approximately 6 weeks old) and weight (230 ± 10 g) were anesthetized intraperitoneally with 20% urethane (0.6 ml/100 g) and secured on a stereotaxic brain apparatus (Stoelting 51603, USA) to determine the rMT and exclude the possibility of any brain damage in the formal experiment. The height of the auricular and incisor rods was adjusted such that the anterior fontanelle and herringbone were in the same horizontal plane. EMG signals were recorded bilaterally using microelectrodes inserted in the biceps femoris muscle and connected to an electromyography system *via* a 6-pin male connector. A standard EMG pad was also connected to the tail to serve as the ground electrode. The “figure eight” coil was placed horizontally on the vertex of the rat’s head, with the center aligned with the midpoint between the rat’s ears. Using 60% TMS output intensity, the coil was gradually moved to determine the best “hotspot,” which was based on a stable MEP waveform recorded in the contralateral biceps femoris muscle. Output intensity was adjusted until the MEP peak did not increase, and this value was defined as the maximum MEP. Stimulation output was gradually reduced to 43–51% (average 45.6% ± 5.73%), and an MEP with amplitude ≥50 μV could be obtained three out of five times on the contralateral side at the maximum output intensity. In our study, 45% (approximately 1.03 T) of the magnetic stimulator output was confirmed as rMT, which ensures a relatively appropriate and reliable rMT intervention in each group.

#### 2.4.2. Priming rTMS protocol followed by a test iTBS

The present study employed a priming-test design consisting of a “priming” rTMS protocol and a subsequent “test” protocol to investigate the intensity-dependent after-effects of 25-Hz repetitive TMS (rTMS) on LTP in the rat hippocampus. Our previous experiments showed that the stimulation of 100% rMT at 25 Hz for 5 s, with a 30-s intertrain interval, can affect the hippocampal field potential and the amplitude of LTP induced by the subsequent iTBS stimulation. Moreover, Ogiue-Ikeda et al. (2003) investigated the intensity-dependent effect of 25 Hz rTMS on LTP in the rat hippocampus, and Cao et al. (2022) found that 25 Hz rTMS could improve cognitive function of Alzheimer’s disease (AD) model mice. Hence, the stimulation frequency of 25 Hz was selected in the present study. The high-frequency rTMS groups received intensity stimulation at 25 Hz for 5 s, which consisted of five 1-s trains of 25 pulses with a 30-s intertrain interval. An intensity of 60–130% rMT was previously used for high-frequency rTMS (Ayache et al., 2012); thus, we defined three subgroups corresponding to three different levels: low intensity (60% rMT), medium intensity (100% rMT), and high intensity (120% rMT).

In general, low-frequency rTMS induces inhibition of synaptic efficiency. Therefore, the low-frequency rTMS group received continuous stimulation of 1 Hz at 100% intensity of rMT for 2 min (Gersner et al., 2011). It was beneficial to keep the amount of stimulus and time frame similar to that in the high-frequency stimulation groups. This method is beneficial for fixing the duration of stimuli while comparing different frequencies and intensities. The control group received sham TMS, which involved exposure to the same noise produced during the simulated stimulus but was treated with a sham coil without real stimulation.

Next, a “test” intermittent theta-burst stimulation (iTBS) protocol was employed to capture the homeostatic response, which consisted of six trains with 10-s intervals between each train containing six bursts at 5 Hz, and each burst containing three pulses at 400 Hz (Kouvaros and Papatheodoropoulos, 2016; Ostrovskaya et al., 2020). The “test” iTBS protocol was administered after the end of priming rTMS in each group.

### 2.5. Experimental schedule

#### 2.5.1. Experiment 1: detection of the population spike and LTP in the hippocampal PP-DG *in vivo*

##### 2.5.1.1. Placement of recording electrode

Five rats in each group were anesthetized with 20% urethane and placed in a stereotaxic apparatus. After routine disinfection, the skin and subcutaneous tissue of the rat’s head were cut open to expose the skull surface. The soft tissue on the skull surface was detached using hydrogen peroxide to completely expose the bregma and the herringbone seam. The brain stereo-position method was used as described by Bruel-Jungerman et al. (2006). A bipolar, 125-mm concentric stimulating electrode was placed in the PP (coordinates: 7.5 mm posterior to bregma, 4.2 mm lateral to the midline, depth of 3.5 mm). A glass micropipette recording electrode was lowered into the DG of the dorsal hippocampus (coordinates: 3.5 mm posterior to bregma, 2.0 mm lateral to the midline) until the maximal PS response was observed (depth: 3–4 mm) (Levkovitz et al., 1999).

##### 2.5.1.2. PS of PP-DG *in vivo*

The optimal recording position for the PP-DG stimulus used the paired-pulse parameters to identify whether PS response was derived from the PP-DG in the hippocampus (McNaughton and Barnes, 1977). The stimulation intensity was adjusted (0–0.7 mA, 0.05-mA interval) to 100 μs of the biphasic pulse at an interval of 30 s for 10 min to record the input–output curve. The standard stimulus intensity of the test stimuli was sufficient to evoke approximately 50% of the maximum response of the PS amplitude. The characteristic response of hippocampal dentate gyrus (DG) granule cells to perforant path stimulation consists of a positive-going EPSP with a superimposed negative-going field PS. The PS amplitude was measured by averaging the distance from the negative to the positive peak. The baseline PS amplitude (PS_0_) represents the initial state of synaptic excitability (Niu et al., 2009), which was averaged from five successive PS responses with a 30-s interval between each stimulus pair for 20 min.

##### 2.5.1.3. After-effects of the rTMS protocol on the LTP plasticity

**Figure 1:**
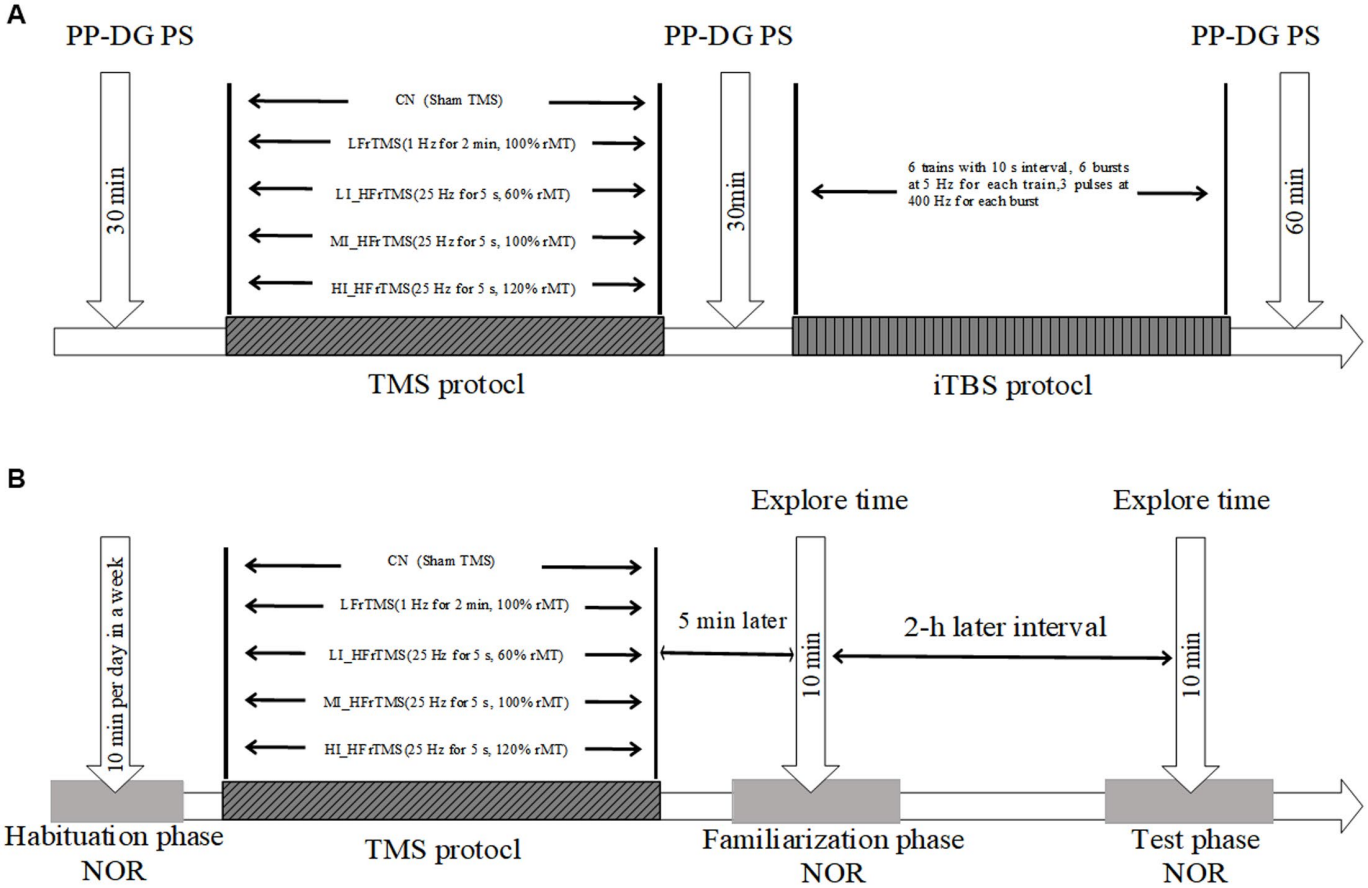
**(A-B)** Experimental time-flow diagram. NOR, novel object recognition.

A priming-test design, a “priming” rTMS protocol and “test” iTBS protocol, was employed to clarify the after-effects of the rTMS protocol for inducing hippocampal excitability. The rat’s head was fixed by hand, the center of the coil was aligned to the rat’s herringbone seam, and the handle was directed outwards in the direction of the longitudinal axis. The PS amplitude after different rTMS (PS_1_) and PS amplitude after iTBS (PS_2_) were recorded continuously for 30 min and 60 min (Figure 1A), with up to ten PS recordings taken at 30-s intervals for 5 min. The maximum and minimum PS were removed, and the mean PS amplitude during 5 min was averaged from five successive PS responses.

LTP was used to detect the synaptic plasticity response in the hippocampus. It was induced after stable baseline recording using iTBS, which was at 80% intensity of rMT stimulation and consisted of six trains with 10-s intervals between each train, containing six bursts at 5 Hz, and each burst containing three pulses at 400 Hz (Kouvaros and Papatheodoropoulos, 2016; Ostrovskaya et al., 2020).

#### 2.5.2. Experiment 2: the effects of the rTMS protocol on NOR task

A priming-test design, a “priming” rTMS protocol and “test” NOR task, was adopted to explore the effects of the rTMS protocol on object recognition. The NOR task was designed by Ennaceur et al. in 1988 (Ennaceur and Delacour, 1988). Briefly, the NOR procedure consists of three phases: habituation, familiarization, and testing. The first week included the habituation phase: each animal was allowed to freely explore the open-field box without any object for 10 min. On the eighth day (familiarization phase), the subject was placed into the open-field arena facing away from two objects and allowed to explore them for 10 min. The exploration time was measured for each of the objects. After a delay of 2 h, the actual testing phase took place: each subject was allowed to explore two different objects (one of them identical to those presented during the familiarization phase, and the other one a novel object) for 10 min. The time spent exploring the novel and familiar objects was recorded by videotaping (Figure 1B).

**Figure 2:**
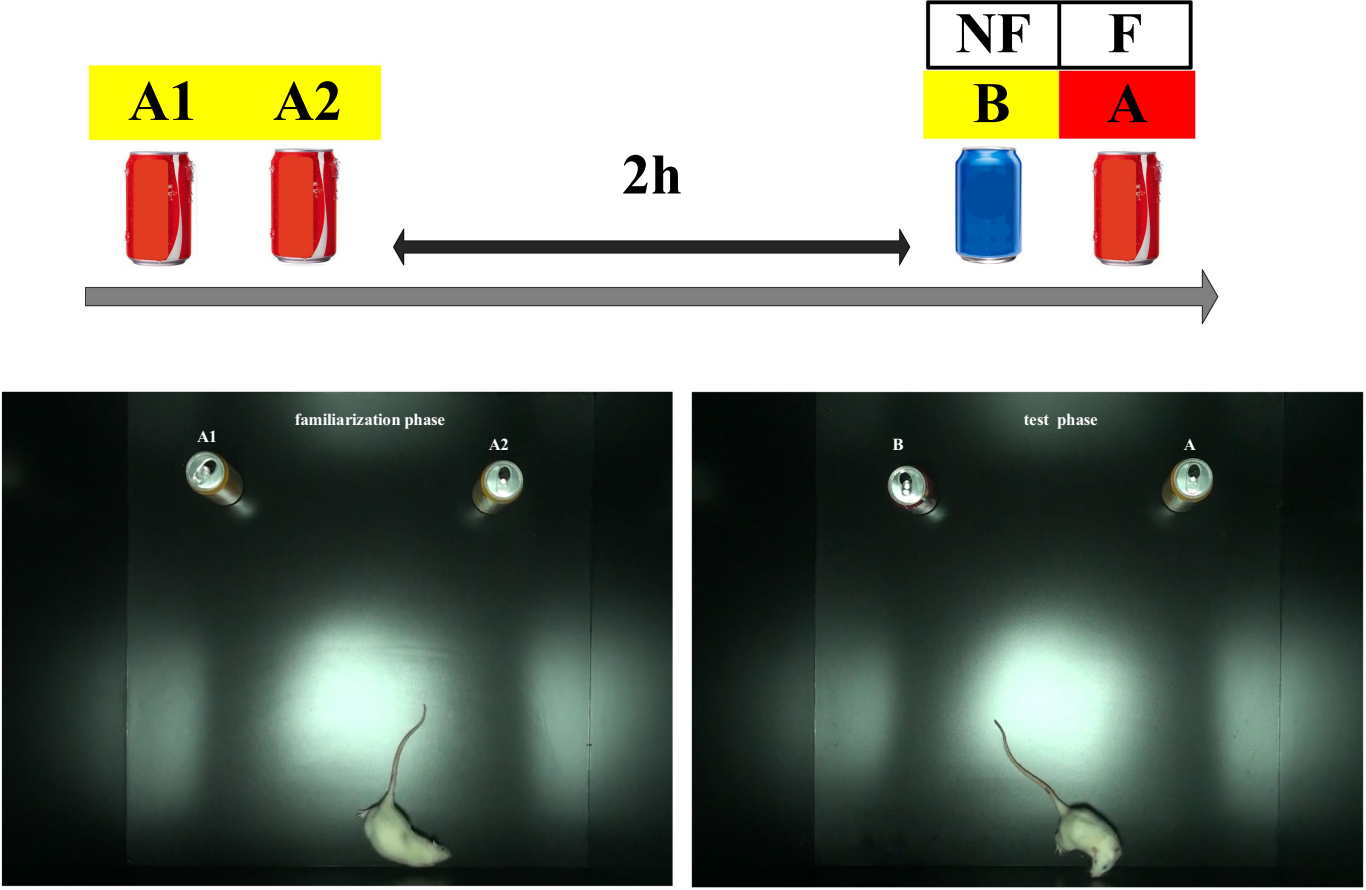
Novel object recognition test pattern diagrams.

The behavioral test was conducted in a quiet environment. Soda cans of different colors were chosen as stimuli owing to their appropriate height and weight, which prevented the subjects from climbing up on them or moving them (Pereira et al., 2014). The rats had never been exposed to these particular objects prior to the NOR task. Objects A or B could be randomly replaced during the familiarization phase, and their position could be randomly changed during the test phase (Figure 2). The box and the objects were cleaned with 75% ethanol after each test to eliminate potential odor cues. Exploration behavior was defined as the time spent sniffing with the nose or whiskers at a distance of less than 2 cm in front of the object or with the front paws touching the object. Turning around or sitting near the object was not considered exploration time. Subjects were excluded from the analyses if they failed to spend at least 1 s exploring each object during the test phase (Sanderson et al., 2011).

### 2.6. Statistical analysis

The LTP amplitudes, representing the capacity for synaptic plasticity in the hippocampus, were calculated using the ratio of the mean PS amplitudes (Niu et al., 2009) for 60 min post-TBS (PS_2_) compared with the pre-tetanus baseline (PS_0_) and expressed as mean ± standard error of mean %. LTP induction was defined as a sustained amplitude response for more than 60 min that reached levels of 130% of the normalized baseline values (Mulder et al., 1997). The time spent exploring each of the two identical objects was designated as (a1) and (a2), and the total time (e1) spent exploring both objects during the familiarization phase was therefore e1 = a1 + a2. Side preferences were tested by comparing a1 and a2 within each group during the familiarization phase. The time spent exploring the familiar (A) and the novel object (B) during the test phase was used to calculate the total time exploring both objects during this phase (e2). Differences between the groups based on the total time spent exploring both objects during the familiarization and the testing phase were evaluated by estimating an index of habituation (h1) using the following formula: h1 = e1-e2. The preference for the novel object was calculated as a discrimination index, which was the ratio of the time spent exploring the novel object divided by the total time spent exploring during the testing phase (i.e., iB = novel/[novel + familiar] = B/e2) (Sanderson et al., 2011). Within-group comparisons were performed using paired Student’s t-test. Between-group comparisons were performed by one-way analysis of variance and post-hoc comparisons. Statistical significance was set at a value of *p* of <0.05. All statistical analyses were performed using SPSS 25.0 (IBM, Armonk, NY, USA).

## 3. Results

### 3.1. Experiment 1: effect of the rTMS protocol on iTBS-induced LTP in the hippocampal PP-DG

**Figure 3:**
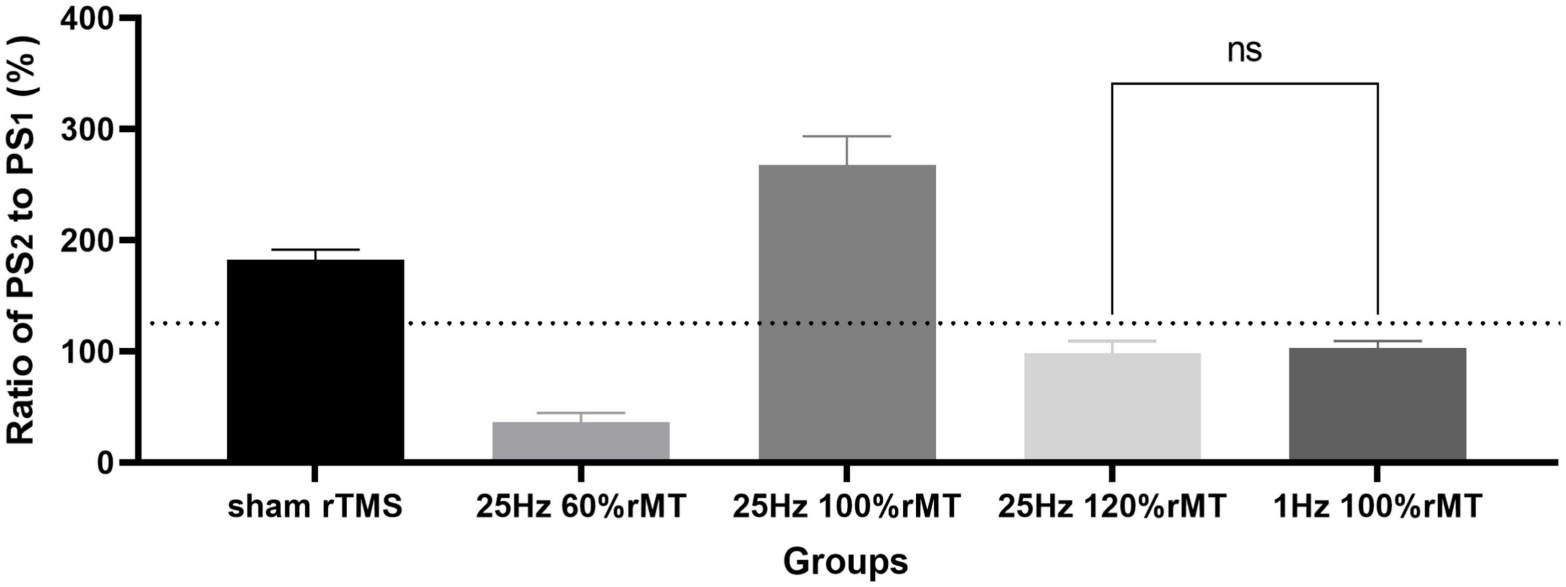
Effects of different repetitive transcranial magnetic stimulation protocols on long-term potentiation generation. “ns” represents “no significance” and the horizontal dashed line represents the level of 130% ratio.

In the control group, in which sham rTMS was induced, the ratio of PS2 amplitude normalized to baseline PS1, namely the PS ratio, was 182% ± 9.4% at 60 min following TBS. The PS ratio was ranked as 25 Hz 100% rMT (267.78% ± 25.71%) > sham rTMS (182 ± 9.4%) > 1 Hz 100% rMT (102.69% ± 6.64%) > 25 Hz 120% rMT (98% ± 11.3%) > 25 Hz 60% rMT (36% ± 8.5%). Significant differences were observed among the groups [*F*(4,20) = 201.5, *p* < 0.001]. No significant difference was found between 25 Hz 120% rMT and 1 Hz 100% rMT (*p* = 0.446); other pairwise comparisons were statistically significant (*p* < 0.001). Only the sham rTMS group and 25 Hz 100% rMT group showed significant enhancement of the amplitude ratio by more than 130% of the baseline following 60 min of iTBS completion, demonstrating successful induction and maintenance of LTP during the 60-min recording period (see Figure 3).

### 3.2. Experiment 2: effect of the rTMS protocol on object recognition

During the familiarization phase, no significant side preference was noted in the sham rTMS (*t* = −1.967, *p* = 0.097), 1 Hz 100% rMT (*t* = −2.268, *p* = 0.073), 25 Hz 60% rMT (*t* = −1.107, *p* = 0.33), 25 Hz 100% rMT (*t* = −0.043, *p* = 0.967), or 25 Hz 120% rMT (*t* = 0.507, *p* = 0.627) groups. The time spent exploring the identical objects was comparable within each group (*p* > 0.05).

**Figure 4:**
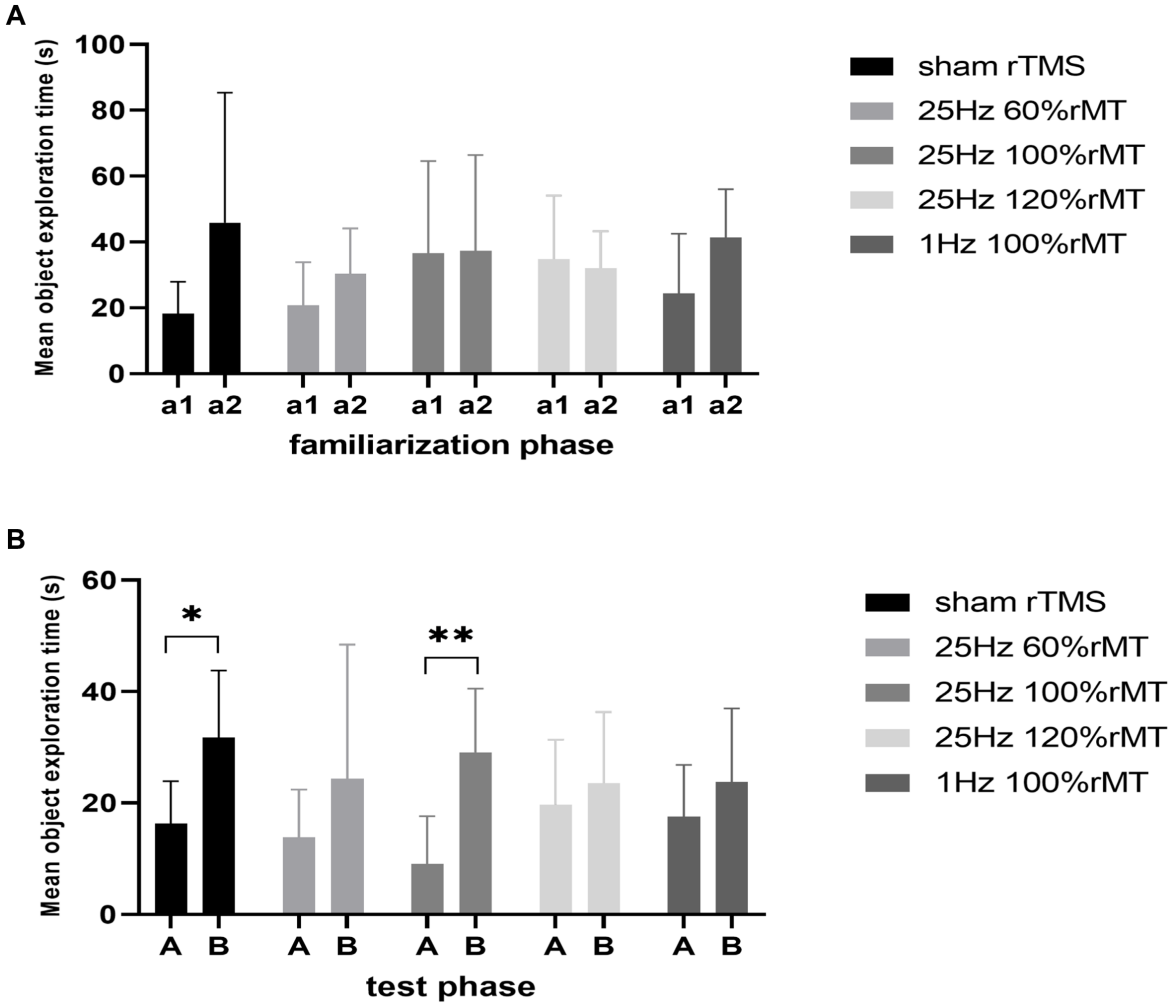
Effect of transcranial magnetic stimulation protocols on time spent in exploring objects. **(A)** a1 and a2 represent familiar objects on the left and right sides, respectively. **(B)** A and B represented familiar and novel objects, respectively. *p < 0.05, **p < 0.01.

During the testing phase, the within-group analysis showed that more time was spent exploring the novel object than the familiar object in the sham rTMS (*t* = −3.441, *p* = 0.014) and 25 Hz 100% rMT (*t* = −6.009, *p* = 0.001) groups. In other words, the subjects spent less time exploring the familiar object than the novel object during the testing phase, indicating that rats in the sham rTMS and 25 Hz 100% rMT groups still retained long-term recognition memory for the familiar object 2 h after having been first exposed to it. No significant difference in exploration time between the familiar and novel object was observed in the 1 Hz 100% rMT (*t* = −1.544, *p* = 0.073), 25 Hz 60% rMT (*t* = −1.493, *p* = 0.21), and 25 Hz 120% rMT (*t* = −0.831, *p* = 0.433) groups during the test phase, revealing an impairment in recognition memory (see Figure 4).

In terms of differences in the total exploration time between the familiarization and the testing phase, no significant differences in the habituation index were observed [*F*(4,29) = 0.534, *p* = 0.712]. The total time spent exploring the objects used as stimuli did not decrease from the familiarization phase to the test phase, suggesting that the rats were equally familiar with their surroundings in both phases and that their physical strength and motivation to explore were not affected by the experimental setup.

**Figure 5:**
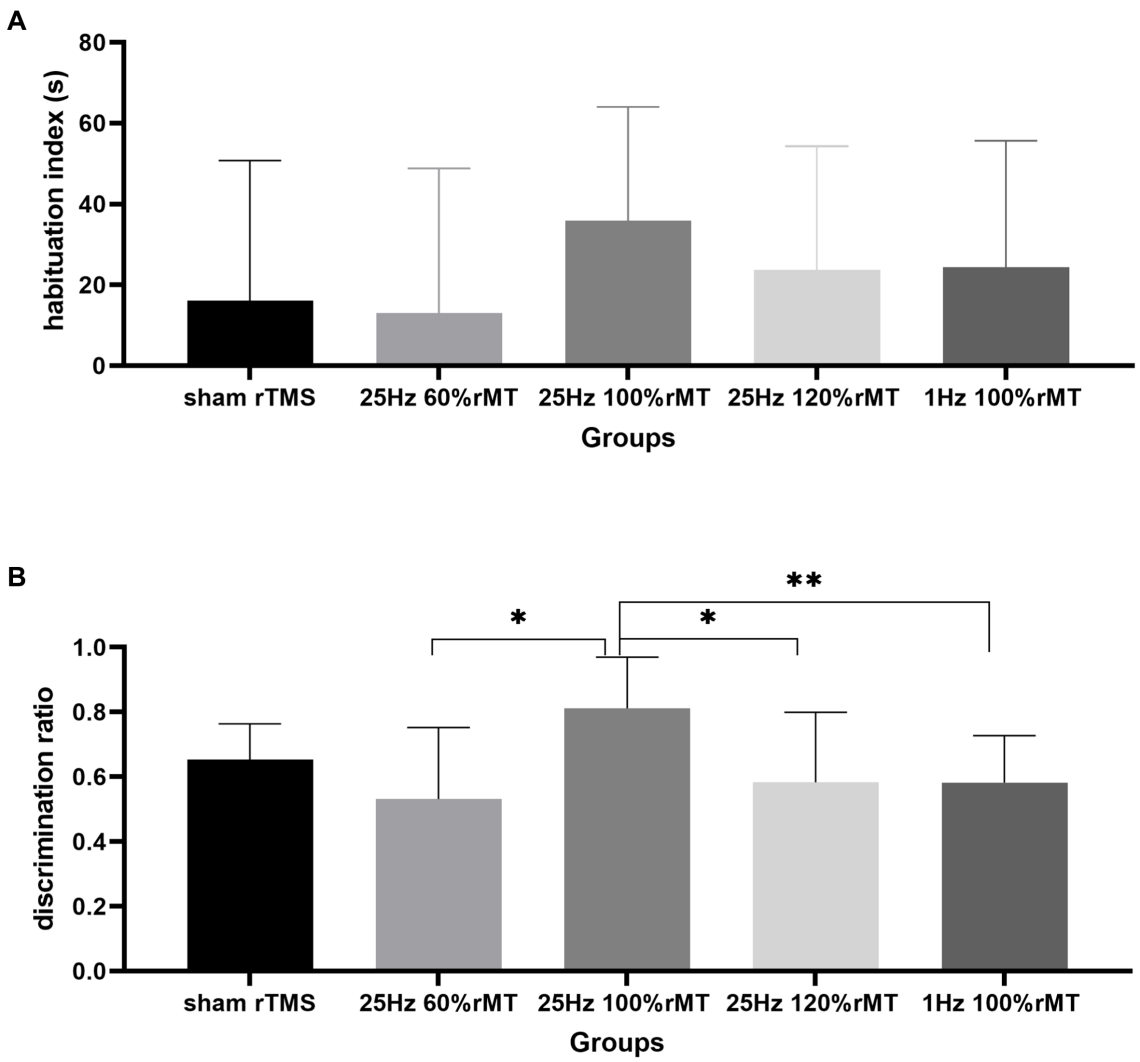
Effect of transcranial magnetic stimulation protocols on the habituation index and discrimination index in exploring objects. **(A)** The habituation index represents the desire to explore objects. **(B)** The discrimination index represents the recognition memory of the familiar object. *p < 0.05, **p < 0.01.

**Figure 6:**
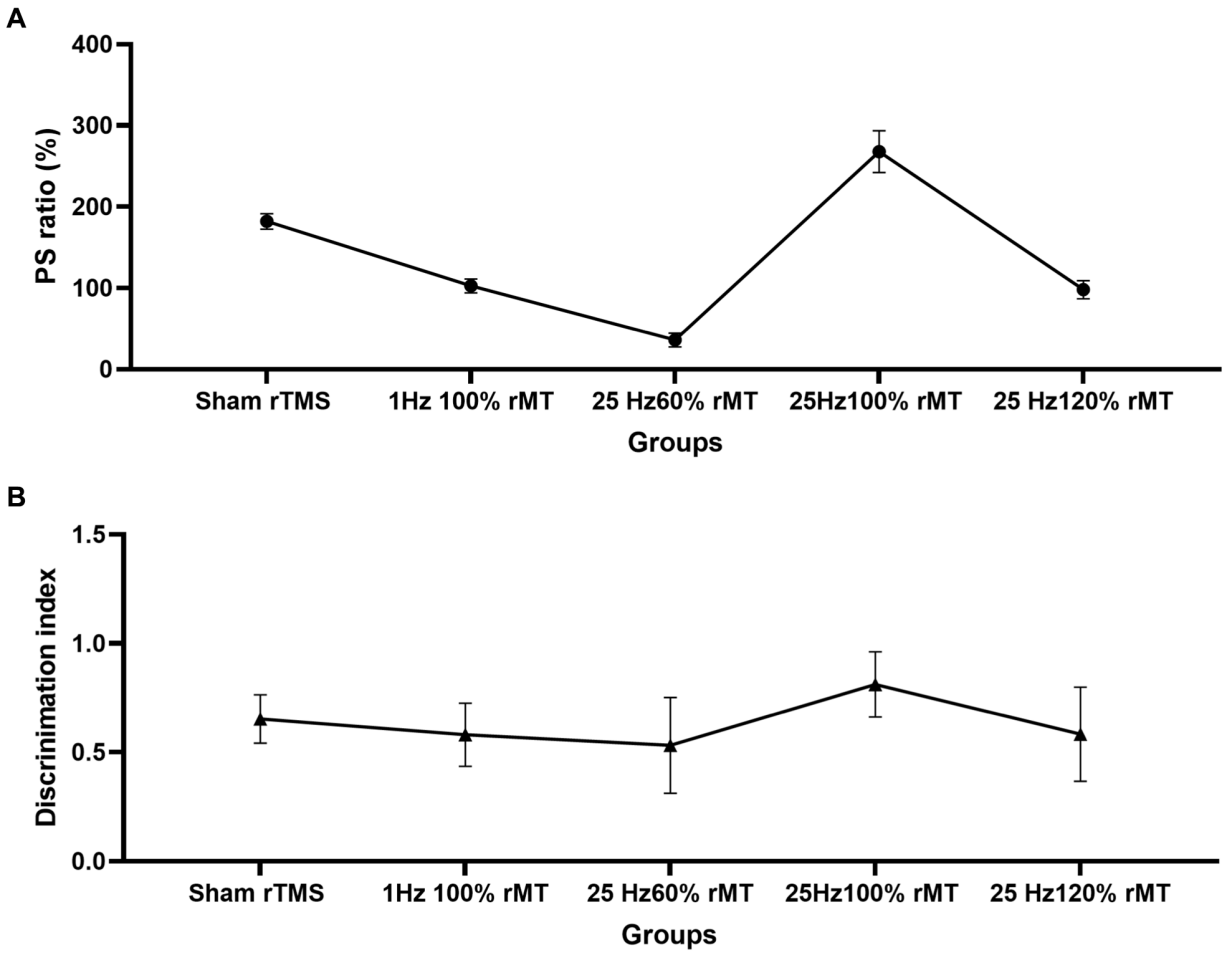
Effect of transcranial magnetic stimulation protocols on the neurophysiologic and behavioral parameters. **(A)** Intensity-dependent effects of rTMS on LTP induction in the hippocampus. **(B)** Intensity-dependent effects of rTMS on the discrimination index of the novel object in the NOR task.

The discrimination index between the groups were significantly different [*F*(4,29) = 2.817, *p* = 0.043]. The discrimination index was ranked as 25 Hz 100% rMT (0.812 ± 0.158) > sham rTMS (0.653 ± 0.111) > 25 Hz 120% rMT (0.583 ± 0.216) > 1 Hz 100% rMT (0.581 ± 0.145) > 25 Hz 60% rMT (0.532 ± 0.220). In the between-group analysis, there were significant differences between the 25 Hz 100% rMT and the 25 Hz 120% rMT (*p* = 0.015), 25 Hz 60% rMT (*p* = 0.006), and 1 Hz 100% rMT groups (*p* = 0.03). There was no difference between sham rTMS and 25 Hz 100% rMT rats (*p* = 0.092). Only the exposure to 25 Hz 100% rMT could significantly enhance memory retention of the familiar object in our experimental subjects (see Figure 5). Furthermore, the discrimination index during the NOR testing phase was calculated as the ratio of the time spent exploring the novel object divided by the total time spent exploring and was found to have a moderate positive correlation (*r* = 0.457, *p* = 0.006), with PS ratio representing the capacity for synaptic plasticity in the hippocampus. Additionally, it was observed that rTMS-induced after-effects on both neurophysiologic and behavioral parameters had a similar trend, suggesting that the intensity-dependent effects of rTMS on LTP induction in the hippocampus were reflected in the performance of the behavioral task (see Figure 6).

## 4. Discussion

We investigated the after-effects of several rTMS intensities on hippocampal plasticity. Our results revealed that 25 Hz rTMS at 100% rMT facilitated iTBS-induced LTP-like plasticity, whereas both 60% rMT and 120% rMT inhibited hippocampal excitability. This was consistent with the inhibitory effect of 1 Hz 100% rMT on the capacity of iTBS-induced LTP. Second, the PS ratio results from the electrophysiological examination were consistent with those of the discrimination index from the NOR test. Thus, we demonstrate here for the first time that high-frequency (25 Hz) rTMS can exert inverted (V-shaped) bidirectional modulation effects on hippocampal plasticity. High-frequency rTMS had a non-linear and intensity-dependent effect on hippocampal plasticity, involving the two forms of the metaplasticity mechanism. Furthermore, the after-effect of high-frequency rTMS with different intensity parameters on the memory recognition ability was positively correlated with those on LTP induction in the hippocampus *in vivo*.

### 4.1. After-effects of rTMS on induced LTP-like plasticity

Numerous studies have demonstrated that rTMS promote cortical excitation according to increasing stimulus intensity. For example, Huang and Rothwell found that MEPs were enhanced after 50 Hz rTMS over the human motor cortex at 70% or 80% active motor threshold (aMT), but not at 50% aMT (Huang and Rothwell, 2004). 3 Hz TMS at 75, 100, and 125% rMT was applied to the primary motor cortex non-linearly with increasing MEP amplitude to healthy volunteers; the rise in the MEP amplitude rate was greater for rMT above 100% (Fox et al., 2006). Both 2 Hz and 6 Hz stimulation at 70% aMT had no effect and that at 80% aMT reduced the magnitude of the MEPs; however, 90% aMT contributed to significant MEP facilitation in some participants and motor cortical excitability inhibition in others (Todd et al., 2006). However, our findings revealed that the application of a high-frequency (25 Hz) rTMS was characterized by an inverted (V-shaped) bidirectional modulation effect on hippocampal plasticity. This resulted in a greater PS ratio (LTP induction) in the 100% rMT (267.78 ± 25.71%) group than those from the 60% rMT (36 ± 8.5%) and 120% rMT (98 ± 11.3%) groups. In contrast with the findings of Todd et al. (2006), in which high-frequency stimulation (at intensities above 90% aMT) partly favored excitatory effects and those below 90% aMT had completely inhibitory effects, we found that high-frequency stimulation at 60% rMT was associated with inhibitory effects. Moreover, we found that high intensity of rTMS was not always associated with excitation: 100% rMT was facilitatory, but 120% rMT had inhibitory effects. Furthermore, we discovered the PS ratio to be greater in the 1 Hz 100% rMT (102.69 ± 6.64%) group than those in the 25 Hz 60 and 120% rMT groups, although this was not sufficient to induce LTP plasticity. Hippocampal excitability is therefore seemingly inconsistent with what has been described in the cortex, where low-frequency rTMS (<1 Hz) decreases and higher-frequency rTMS (>5 Hz) increases cortical excitability.

Hence, our viewpoint stresses that the modulation of rTMS on LTP in the rat hippocampus is based on the appropriate stimulus intensity but is not necessarily proportional to the intensity or the frequency of the excitation. Ogiue-Ikeda et al. (Ogiue-Ikeda et al., 2003) had previously found that LTP in the rat hippocampus was enhanced at 0.75-T intensity (rTMS parameters: 10 1-s trains of 25 pulses with a 1-s intertrain interval) but suppressed by 1.25-T intensity, with no change observed at 0.5-T and 1.0-T. The overall conclusion that can be drawn from both studies is that high-frequency rTMS does not induce a linear increase in LTP with the increase in output intensity but is instead characterized by a V-shaped bidirectional modulation of LTP plasticity.

### 4.2. Association between LTP and memory recognition

NOR memory is hippocampus-dependent (Zhang et al., 2021). The behavioral results related to the NOR test were consistent with the neurophysiology results in this study. Recognition memory was reflected by the discrimination indexes of each experimental group. In Figure 5, the discrimination index ranking according to the rTMS level was as follows: 25 Hz 100% rMT > sham rTMS >25 Hz 120% rMT > 1 Hz 100% rMT > 25 Hz 60% rMT. In Figure 3, the ranking for the LTP ratios was, in turn, 25 Hz 100% rMT > sham rTMS >1 Hz 100% rMT > 25 Hz 120% rMT > 25 Hz 60% rMT. No between-group differences were observed between the 1 Hz 100% rMT and 25 Hz 120% rMT groups. As depicted in Figure 6, the aftereffects of rTMS on neurophysiological and behavioral parameters exhibited a similar trend, indicating that the intensity-dependent effects of rTMS on hippocampal LTP are reflected in the recognition memory performance of the NOR task.

Hippocampal LTP has been considered to represent a synaptic model of memory, associated with some specific forms of behavioral learning (Bliss and Collingridge, 1993). Numerous studies have demonstrated the association of LTP with learning and memory in animals. The earliest landmark studies from 1979 which applied a multi-session high-frequency stimulation protocol to induce LTP *in vivo* in the rat DG, found that aged individuals exhibited lower LTP magnitudes along with slower spatial memory acquisition and accelerated forgetfulness. Furthermore, the LTP magnitude correlated with the task performance in both young and aged rats (Barnes, 1979; Barnes and McNaughton, 1985). Since then, the correlation between the LTP characteristics and memory performance has been widely explored. Rat exposure to environmental levels of lead resulted in learning and memory impairment closely associated with a decrease in LTP induction ability (Lasley et al., 1993; Gilbert et al., 1996). The amplitude of LTP induction was significantly higher in rats with better learning ability than in controls (Wang et al., 2007). Some studies found that Wistar rats with stable LTP during recordings of 180 min following TBS showed increased novel object exploration time, suggesting that LTP maintenance is associated with long-term memory retention (Guimaraes et al., 2018). Proechimys rats showed increased LTP induction but not maintenance, with the potentiation decaying over time and reaching basal levels 90 min after TBS. These rats spent a similar amount of time exploring familiar and novel objects, suggesting long-lasting memory impairment in addition to the LTP decay (Guimaraes et al., 2018). In the present study, we found sham rTMS and 25 Hz 100% rMT induced higher LTP (>130% of baseline) for 60 min following iTBS, and the subjects retained the memory of familiar objects for a longer period, which supports the LTP-memory hypothesis that enhanced and suppressed LTP correlated with better and impaired learning and memory capacity, respectively (Dringenberg, 2020).

Similarly, the association between LTP and memory recognition was found in the *in vivo* demonstration of synaptic impairment in AD patients. Positron emission tomography of synaptic vesicle glycoprotein 2A has revealed a widespread synaptic loss in the brains of AD patients, demonstrating that alteration of the LTP mechanism is associated with memory impairment (Francesco and Koch, 2021). The investigation of rTMS stimulation parameters in rats has important implications for improving synaptic function. For instance, 25 Hz 100% rMT facilitated LTP induction and maintenance, which may be an effective therapeutic approach to counteract cognitive impairment in pathology. Therefore, it is necessary to identify potential therapeutic targets by investigating detailed rTMS stimulation parameters, including frequency and density, in future human research.

### 4.3. Intensity dependence of the rTMS after-effect on LTP and memory

rTMS protocols vary greatly in the frequency and intensity of stimulation. Overall, studies suggest a facilitatory effect of high-frequency rTMS (i.e., ≥5 Hz) and an inhibitory effect of low-frequency rTMS (≤1 Hz) (Chen et al., 1997; Fitzgerald et al., 2006). We observed that the modulatory effect of high-frequency (25 Hz) rTMS on iTBS-induced LTP-like plasticity was bidirectional, being facilitatory in the case of 25 Hz 100% rMT, and inhibitory in cases of 25 Hz 60 and 120% rMT. On the other hand, 1 Hz 100%, 25 Hz 60, and 120% rMT inhibited LTP induction and aggravated memory impairment in the hippocampal PP-DG, whereas 25 Hz 100% rMT promoted LTP plasticity and enhanced the object recognition ability in our subjects.

Metaplasticity is a higher-order form of synaptic plasticity referring to the synaptic activity that primes the ability to induce subsequent synaptic LTP- or long-term depression (LTD)-like plasticity (Karabanov et al., 2015). According to the Bienenstock, Cooper and Munro (BCM) theory (Jedlicka, 2002), the change in the excitability of synapses would inevitably affect the threshold of synaptic plasticity, which is specifically manifested in two different regulation modes. The greater improvement of memory observed in response to the 25 Hz 100% rMT protocol in the present study was consistent with the results of a previous study in which high-frequency rTMS modulated corticomotor inhibition and enhanced the effect of treadmill training in the motor learning (Yang et al., 2013). Form plasticity of gating mechanisms (Ziemann and Siebner, 2008) may be suitable for the interpretation of excitatory priming rTMS and may increase the response to subsequent iTBS-induced LTP-like plasticity, which may provide an effective means to induce transient disinhibition or depolarization and boost recognition memory. Furthermore, gating is another mechanism of metaplasticity wherein the priming intervention does not have a homeostatic effect on the subsequent stimulus/training (Karabanov et al., 2015). Similarly, we observed no significant increased effect of 25 Hz 60% rMT on LTP-like plasticity response to subsequent facilitatory iTBS, with memory recognition impairment. This could be attributed to a strong inhibitory effect resulting from high-frequency and low-intensity TMS pulses (Arai et al., 2009). Thus, inhibitory priming rTMS would inhibit the response to subsequent facilitatory iTBS (Todd et al., 2009).

Interestingly, the fact that 25 Hz 120% rMT had an inhibitory effect on iTBS-induced LTP-like plasticity was unexpected. Also, we observed that the in 25 Hz 120% rMT group the discrimination index was low in the NOR task, indicating impaired recognition memory. It has been well-established that synaptic modifications can be reversed by subsequent stimuli, as demonstrated by the reversal of hippocampal LTP in rats upon entry into a novel environment (Zhou and Poo, 2004). We speculated the phenomenon is consistent with the compensatory change in homeostatic plasticity theory, which suggests that facilitatory priming TMS may weaken or reverse the effect of subsequent facilitatory test/intervention measures. This has also been described in another study that demonstrated a reversal in the homeostatic excitability effect using the same consecutively applied non-invasive transcranial brain stimulation protocol (Muller et al., 2007). The previous fundamental studies revealed that the form of homeostatic plasticity emphasized maintaining stabilized neural activity in a meaningful physiological range (Karabanov et al., 2015) to avoid LTP saturation (Moser et al., 1998). Hence, the increase in LTP amplitude was limited, and memory retention declined based on the LTP that correlated with common neural memory mechanisms in the hippocampus, which often required the activation of N-methyl D-aspartate receptors and their intracellular signaling (Roman et al., 1999). Considered a reference of moderate LTP amplitude induced by only iTBS in sham rTMS, increased LTP was induced if primed by 25 Hz 100% rMT but LTP decreased if primed by 25 Hz 60 and 120% rMT, and even 1 Hz 100% rMT. However, the reason for the LTP amplitude of 25 Hz 120% rMT showing no difference from that of 1 Hz 100% rMT, all of which surpassed the 25 Hz 60%, remains unclear.

In summary, the results of this study indicate that the priming protocol characteristics (i.e., intensity and frequency) are critically important for the induction of metaplasticity. We speculate that metaplasticity ensures a safe threshold zone. In cases in which gating plasticity plays a positive regulatory role in excitatory priming rTMS, it would facilitate the response to the subsequent protocol. If the ceiling or floor for homeostatic plasticity is exceeded, the metaplasticity would then provide negative feedback on the inhibitory or facilitatory priming rTMS, weakening or reversing the effect of the subsequent inhibitory or facilitatory protocol to guarantee normal synaptic activity.

### 4.4. Limitations

First, only the intensities of 60, 100, and 120% rMT were used in this study to investigate the effect of 25-Hz high-frequency rTMS stimulus. An intensity range of 60–130% rMT is the most commonly used parameter for high-frequency rTMS in a previous report (Ayache et al., 2012); however, due to time and expense constraints, 60, 100, and 120% rMT were selected as they are clinical commonly used parameters. Fixed parameters of rTMS stimulation frequency and intensity and varying stimulation time also have been tested and will be described in other articles. Second, the study design did not allow us to explore which specific mechanism was involved in the effect we describe. The use of specific antagonists or knockout models for saturating/occluding LTP might provide additional insight into the molecular mechanisms underlying the impaired learning and task acquisition reported. In addition, the exploration index correlated with the amplitude of LTP induction, but there was no significant difference between each of the experimental groups and the group receiving sham TMS. This could be because we only selected a 2-h interval for the NOR test. Ennaceur and Delacour (1988) and Ennaceur and Meliani (1992) found that dose-dependent changes in piracetam-induced changes in memory ability only became evident after longer intervals. Rogel-Salazar et al. (2013) observed that the recognition indexes in rats that had received transcranial focal stimulation were different according to the delay in the evaluation of short- and long-term memory. Thus, we cannot exclude the possibility that the difference between the experimental groups could have been significant if the interval between the familiarization and the testing phases had been extended. Although limited by its design, this trial may contribute another perspective on the application of rTMS for the treatment of memory impairment.

Furthermore, it is important to note that direct stimulation of the hippocampus is not possible in humans, unlike in animal models. However, the cerebellum and dorsolateral prefrontal cortex (DLPFC) have been identified as potential brain areas that are interconnected with the hippocampus, especially in patients with AD (Di Lorenzo et al., 2020; Yao et al., 2022). In addition, the stimulus sequence of the rTMS parameter on plasticity was related to spike-timing-dependent plasticity (STDP) in patients investigating the connections between the cortico-cortical cortex. In particular, STDP was altered in AD patients and this might represent a critical event in memory impairment (Di Lorenzo et al., 2018). Therefore, future animal model studies about the after-effect of rTMS parameters on LTP and memory should be conducted on the same regions that are stimulated in humans. This could help facilitate the development of new potential therapeutic strategies to modulate neural activity in patients with cognitive impairment.

## Data availability statement

The original contributions presented in the study are included in the article/Supplementary materials, further inquiries can be directed to the corresponding authors.

## Ethics statement

The animal study was reviewed and approved by the Institutional Animal Care and Use Committee of the Faculty of Medicine, Xiamen University, China (approval no. SYXK(min)-2018-0009).

## Author contributions

SC and XH conducted the study and including data collection. XW participated in data collection as a member. SC conducted data analysis, prepared the manuscript, and prepared the manuscript draft with important intellectual input from XH, JH, and JZ. JH and JZ designed the study. All authors contributed to the article and approved the submitted version.

## Funding

This research was funded by the Ministry of Science and Technology of Fujian (Grant numbers: 2020D025 and 2020CXB052) to XH, and the Youth Innovation Project from the Ministry of Science and Technology of Fujian (grant number: 2022D010) to SC.

## Conflict of interest

The authors declare that the research was conducted in the absence of any commercial or financial relationships that could be construed as a potential conflict of interest.

## Publisher’s note

All claims expressed in this article are solely those of the authors and do not necessarily represent those of their affiliated organizations, or those of the publisher, the editors and the reviewers. Any product that may be evaluated in this article, or claim that may be made by its manufacturer, is not guaranteed or endorsed by the publisher.
